# Construction and validation of a prognostic signature based on necroptosis-related genes in hepatocellular carcinoma

**DOI:** 10.1371/journal.pone.0279744

**Published:** 2023-02-16

**Authors:** Yue-ling Peng, Ling-xiao Wang, Mu-ye Li, Li-ping Liu, Rong-shan Li

**Affiliations:** 1 Department of Nephrology, Shanxi Provincial People’s Hospital (Fifth Hospital of Shanxi Medical University), Taiyuan, China; 2 Department of Colorectal and Anal Surgery, Shanxi Provincial People’s Hospital (Fifth Hospital of Shanxi Medical University), Taiyuan, China; 3 Department of Ocular Fundus Diseases, Shanxi Eye Hospital, Shanxi Medical University, Taiyuan, China; 4 Department of Ultrasound, First Hospital of Shanxi Medical University, Taiyuan, China; Kimura Hoospital, JAPAN

## Abstract

**Background:**

Necroptosis is a necrotic programmed cell death with potent immunogenicity. Due to the dual effects of necroptosis on tumor growth, metastasis and immunosuppression, we evaluated the prognostic value of necroptosis-related genes (NRGs) in hepatocellular carcinoma (HCC).

**Methods:**

We first analyzed RNA sequencing and clinical HCC patient data obtained to develop an NRG prognostic signature based on the TCGA dataset. Differentially expressed NRGs were further evaluated by GO and KEGG pathway analyses. Next, we conducted univariate and multivariate Cox regression analyses to build a prognostic model. We also used the dataset obtained from the International Cancer Genome Consortium (ICGC) database to verify the signature. The Tumor Immune Dysfunction and Exclusion (TIDE) algorithm was used to investigate the immunotherapy response. Furthermore, we investigated the relationship between the prediction signature and chemotherapy treatment response in HCC.

**Results:**

We first identified 36 differentially expressed genes out of 159 NRGs in hepatocellular carcinoma. Enrichment analysis showed that they were mainly enriched in the necroptosis pathway. Four NRGs were screened by Cox regression analysis to establish a prognostic model. The survival analysis revealed that the overall survival of patients with high-risk scores was significantly shorter than that of patients with low-risk scores. The nomogram demonstrated satisfactory discrimination and calibration. The calibration curves validated a fine concordance between the nomogram prediction and actual observation. The efficacy of the necroptosis-related signature was also validated by an independent dataset and immunohistochemistry experiments. TIDE analysis revealed that patients in the high-risk group were possibly more susceptible to immunotherapy. Furthermore, high-risk patients were found to be more sensitive to conventional chemotherapeutic medicines such as bleomycin, bortezomib, and imatinib.

**Conclusion:**

We identified 4 necroptosis-related genes and established a prognostic risk model that could potentially predict prognosis and response to chemotherapy and immunotherapy in HCC patients in the future.

## Introduction

Hepatocellular carcinoma is the most common form of primary liver cancer as well as the fourth leading cause of cancer-related death in the general population [1]. Hepatocellular carcinomas comprise a heterogeneous group of malignant diseases, and the prognosis of individuals varies widely [2]. The most significant risk factors for HCC are cirrhosis (chronic liver damage caused by fibrosis), hepatitis B virus (HBV) infection, hepatitis C virus (HCV) infection, alcohol abuse and metabolic syndrome, and patients with these risk factors have worse outcomes and lower overall survival [3, 4]. Despite significant improvements in treatments such as surgery, ablation, transarterial embolization, radiation therapy and chemotherapy, a substantial number of patients suffer from recurrence and metastasis [5, 6]. HCC has an exceedingly dismal prognosis, with a 5-year average survival rate of less than 10%. However, the 5-year survival rate for patients with early metastasis and recurrence can be markedly lower [7, 8]. Rather than including genetic characteristics, the existing classical prognostic model of liver cancer only incorporates TNM tumor stage, histological grading, and clinical characteristics for assessing the prognosis of hepatocellular cancer and, which may lead to inaccurate assessment of patient prognosis. Molecular targeted therapy has more promising potential for liver cancer treatment in the near future [9–12]. Therefore, new molecular markers for prognosis prediction and therapeutic targets in hepatocellular cancer are urgently needed.

Necroptosis is a newly discovered type of necrotic cell death with a mechanistic similarity to apoptosis and a morphological similarity to necrosis [13]. According to recent studies, necroptosis has been suggested to be involved in cancer biology, including tumorigenesis, cancer metastasis, cancer immunology, and cancer subtypes [14, 15]. Necroptosis has also been identified to be both a friend and a foe of cancer, with its dual effects on tumorigenesis, progression and metastasis; moreover, necroptosis is intricately linked to apoptosis and autophagy in biological processes [16–19]. A large number of studies have reported a close link between necroptosis and hepatocellular carcinoma [20–22], and necroptosis-related genes (NRGs) might be useful as prognostic biological markers for HCC patients. For example, Tang, R. *et al*. reported that necroptosis combined with immune checkpoint inhibitors (ICIs) exhibited synergistic anticancer efficacy, even in ICI-resistant tumors [23]. However, the clinical value of necroptosis has not been tested in HCC patients. Therefore, it is of great significance to find new molecular markers of necroptosis for precise prognosis prediction and treatment.

In this study, for the first time, we comprehensively analyzed the potential clinical value of necroptosis-related genes as prognostic biomarkers in HCC and established a new hepatocellular carcinoma prognostic model to predict sensitivity to immunotherapy and chemotherapy. Ultimately, a 4-gene-based prognostic risk model was developed, which provided more accurate predictive value in for HCC patients than other models.

## Materials and methods

### Data acquisition and differential expression analysis

The 159 NRGs were collected from the Kyoto Encyclopedia of Genes and Genomes (KEGG) database in November 2021. The 159 NRGs are shown in S1 Table. All expression data and clinical characteristics data of HCC patients were downloaded and extracted from The Cancer Genome Atlas (TCGA). These included data on 374 HCC tissues and 50 nontumor control tissues. An independent external dataset (ICGC-LIRI-JP) that contained 232 HCC tissues and the corresponding clinical follow-up information served as a validation cohort. The "limma" package was used to perform differential expression analysis (|logFC|>1, FDR < 0.05) on the normal and tumor groups.

### Functional enrichment analysis

Functional and pathway enrichment analyses of differentially expressed NRGs were conducted using the "clusterProfiler" package. The "GOplot" package was used to visualize the KEGG functional pathway analysis data. GO terms and KEGG pathways with p < 0.05 were considered statistically significant.

### Construction of NRG-related prognostic model

The "survival" package was used to perform univariate regression analysis on differentially expressed genes (DEGs). On the basis of candidate prognostic genes, multivariate Cox regression analysis was carried out. Next, the risk signature was developed based on the regression coefficients and gene expression values derived from the multivariate Cox regression analyses. The following formula was used to calculate each patient’s risk score: risk score = (expr gen1×Coef gen1) + (expr gen2×coef gen2) +…. + (expr genen × coef genen). According to the median risk score, the HCC patients were divided into a low-risk group and a high-risk group. Meanwhile, to assess the predictive accuracy of the prognostic signature, ROC (receiver operating characteristic) curves for 1-, 3-, and 5-year survival were constructed, and the AUC (area under the curve) values were calculated with the survival ROC package.

### Development and validation of the nomogram

The survival and rms packages for R were used to generate a nomogram using significant univariate analysis components (age, sex, grade, stage, T stage, N stage, M stage, and risk score). We also used the concordance index (C-index) to assess the model’s prognostic prediction ability. Afterward, a calibration curve was constructed to assess the consistency between actual and predicted survival.

### Immunohistochemistry analysis

Twenty tumor tissues and 10 normal liver specimens representing patients managed between January 2021 and August 2022 were obtained from Shanxi Provincial People’s Hospital. The experiments were approved by the Ethical Committee of the Shanxi Provincial People’s Hospital (No. 2022–264), and written informed consent was signed by each participant. The primary antibodies used were as follows: polyclonal rabbit anti-HSP90AA1 (cat. no. 17856-1-AP; 1:100 dilution; Proteintech); polyclonal rabbit anti-PPIA (cat. no. 10720-1-AP; 1:100 dilution; Proteintech); polyclonal rabbit anti-SQSTM1 (cat. no. 18420-1-AP; 1:100 dilution; Proteintech); and polyclonal rabbit anti-USP21 (cat. no. 17856-1-AP; 1:100 dilution; Proteintech). The slides were immersed in xylene three times for 10 min each and then run through 100%, 90%, 80%, and 70% alcohol for 5 min each. Slides were then rinsed with deionized water before rehydration in phosphate buffered saline (PBS) for 6 min. Samples were incubated with two drops of 3% hydrogen peroxide for 20 min to quench endogenous peroxide activity. Samples were washed with PBS for 15 min, followed by incubation with primary antibody at 4°C overnight. Samples were washed with PBS three times for 5 min each before incubation with secondary antibody for 30 min. After washing three times with PBS, samples were developed using DAB/AEC chromogen solution. The samples were rinsed three times with PBS, rinsed in deionized water, and mounted with hematoxylin. Slides were visualized using an EasyScan 6 system. Images were captured on an EasyScan digital slide scanner (Motic) at 40x magnification. Two pathologists who were unaware of the clinicopathological data independently evaluated the immunostained sections. The intensity of staining was scored as follows: 0 (no staining), 1 (weak), 2 (moderate), and 3 (high). Percentage scores were assigned as follows: 1 (1–25%), 2 (26–50%), 3 (51–75%), and 4 (76–100%). The scores were multiplied to obtain a final score of 0–12. Samples with scores of ≥4 were considered to present high expression, and samples with scores between 0 and 4 were considered to present low expression. Analysis of gene expression patterns in HCC and normal liver tissues was performed via Wilcoxon signed-rank tests in SPSS v.26.0.

### Chemotherapy drug response prediction based on the NRG signature

To evaluate the predictive signature’s significance in predicting chemotherapy sensitivity in HCC, the "pRRophetic" package was used to calculate the half-maximal inhibitory concentration (IC50) of the primary chemotherapeutic agents used in the treatment of HCC patients.

### Prediction of response to immunotherapy

TIDE (tumor immune dysfunction and exclusion) is a computational approach designed by Harvard University to predict the response to immune checkpoint blockade treatment by modeling tumor immune evasion mechanisms. The TIDE website (http://tide.dfci.harvard.edu/) was used in this work to investigate the sensitivity to immunotherapy of different risk groups based on transcriptome profiles of HCC patients from TCGA.

### Statistical analysis

All statistical analyses, including univariate and multivariate Cox regression, ROC curve analysis, and Kaplan-Meier survival analysis, were performed using the R language (version 4.0.3). Except for the special instructions, all statistical tests were bilateral, and P < 0.05 was considered to be statistically significant.

## Results

### Identification of differentially expressed NRGs

**Fig 1:**
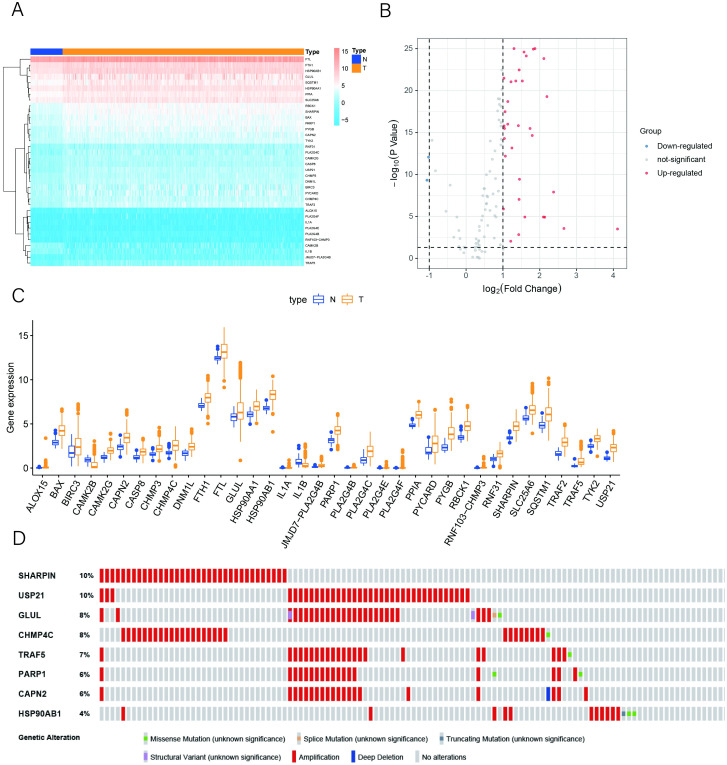
Differential expression analysis. (A) Heatmap of 36 differentially expressed NRGs based on their expression levels. (B) The volcano plot of the differentially expressed NRGs. (C) Boxplot of the expression of necroptosis-related DEGs. N indicates nontumor tissues; T indicates tumor tissues. (D) Mutations in NRGs. A total of 8 genes had a mutation rate ≥3%.

RNA-seq data from 374 tumor tissue samples and 50 nontumor samples were downloaded from TCGA, and the expression values of 132 NRGs were extracted from HCC patient data. According to the criteria for FDR < 0.05 and [log2 (fold change)] >1, we identified 2 downregulated genes and 34 upregulated genes (Fig 1A and 1B). A boxplot was constructed to show the expression patterns of these differentially expressed NRGs between tumor and nontumor tissues (Fig 1C). We further explored the genetic alterations of these genes. As shown in Fig 1D, 8 genes had a mutation rate ≥3% in differentially expressed NRGs, and missense mutation was the most common type of mutation.

### Functional annotation

**Fig 2:**
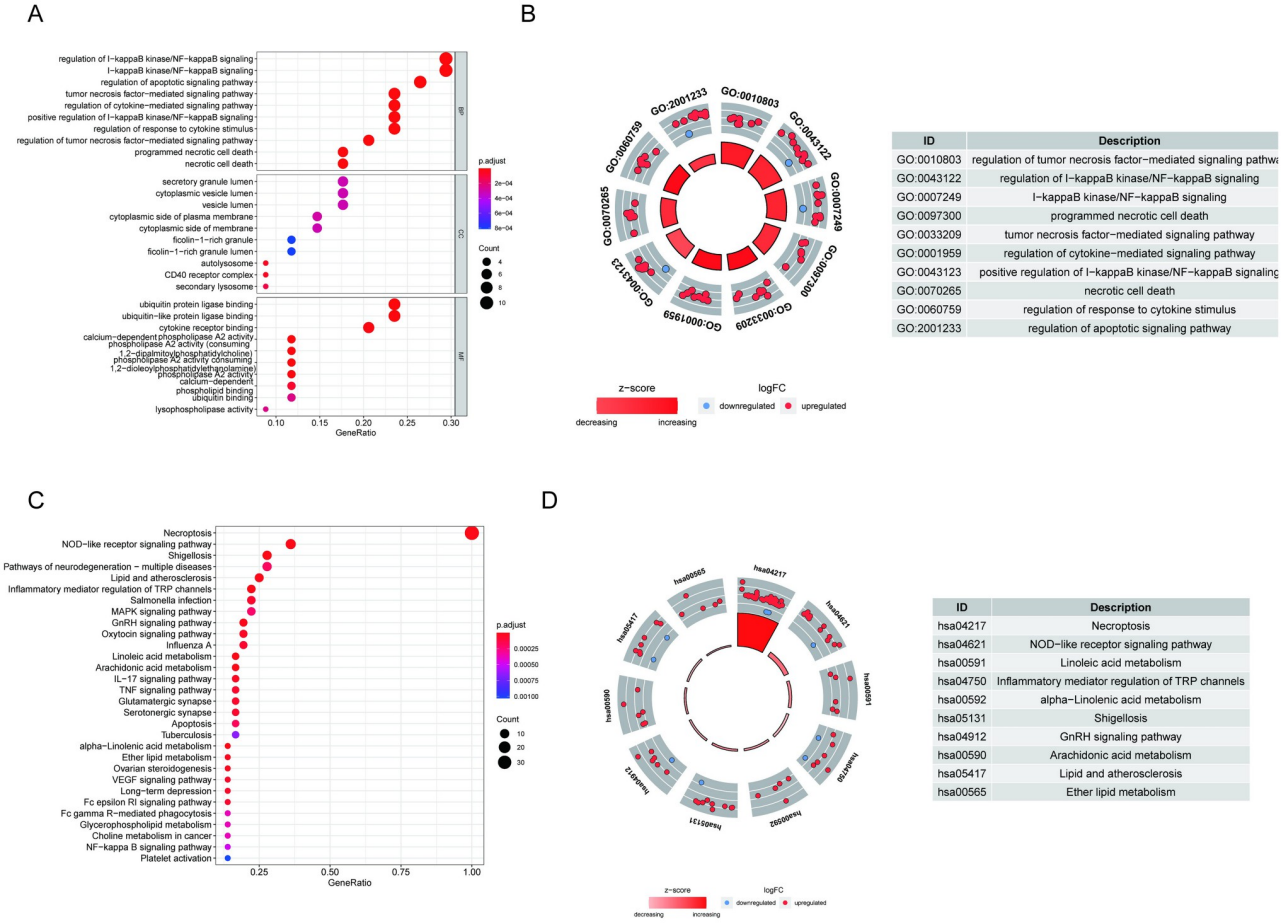
GO and KEGG analysis. (A) GO function enrichment. Biological process, BP; Cellular component, CC; Molecular function, MF. (B) The GO circle shows the scatter map of the logFC of the specified gene. (C) KEGG functional enrichment. (D) The KEGG circle shows the scatter map of the logFC of the specified gene. Blue: downregulated; red: upregulated.

Functional enrichment analysis of the 36 differentially expressed genes was undertaken to investigate the basic signal transduction pathways and biological processes. GO enrichment analysis showed that the biological processes of the differentially expressed genes were mainly involved in the regulation of I−kappaB kinase/NF−kappaB signaling and the regulation of the apoptotic signaling pathway. In the KEGG pathway enrichment analysis, these genes were shown to be notably associated with pathways related to necroptosis, the NOD−like receptor signaling pathway, and shigellosis (Fig 2A–2D).

### Construction of the signature predicting prognosis

**Fig 3:**
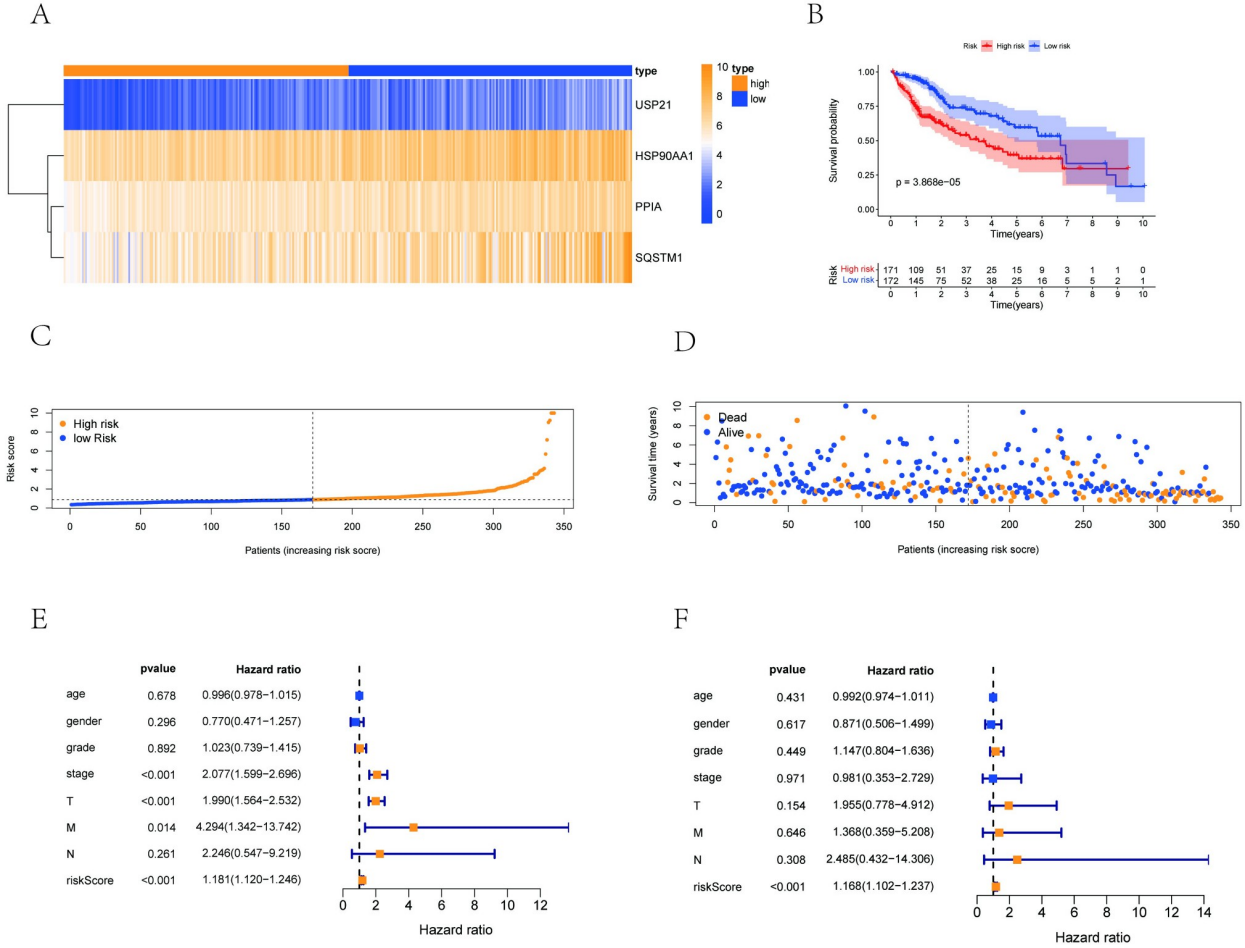
Development of a prognostic index based on NRGs. (A) Heatmap of the expression profile of 4 NRGs (HSP90AA1, PPIA, SQSTM1, USP21). (B) Survival status of patients in the high-risk and low-risk groups. (C) Distribution plot of the risk score of patients. (D) Distribution plot of the survival status of patients. (E, F) The forest plot of univariate and multivariate Cox regression analysis in HCC.

This univariate Cox regression analysis indicated that 7 of the 36 differentially expressed NRGs were strongly significantly associated with survival (p < 0.05, hazard ratio > 1) (S1 Fig). Next, multivariate regression analysis was conducted based on the screened genes above to establish a prognostic risk model, and 4 NRGs capable of predicting HCC clinical outcomes (HSP90AA1, PPIA, SQSTM1, USP21) were identified. A heatmap of these 4 NRGs is shown in Fig 3A. Finally, we established the risk assessment model based on the 4 NRGs. The equation is as follows: Risk Score = 0.0029 * expression level of HSP90AA1 + 0.0086 * expression level of PPIA + 0.0024 * expression level of SQSTM1 + 0.9751 * expression level of USP21. The median value of the risk score was used as a cutoff to distinguish between the high-risk and low-risk groups. A Kaplan–Meier analysis was conducted to determine the predictive performance. According to Fig 3B, the survival rate of patients in the high-risk group was significantly lower than that in the low-risk group. The distribution of the prognostic index and survival status of patients in different groups is also displayed in Fig 3C and 3D. Meanwhile, we verified the independent predictive value of the necroptosis-related signature for OS using univariate and multivariate Cox regression analyses. As shown in Fig 3E, the univariate Cox analysis revealed that the risk score, tumor stage, T stage, N stage and M stage were significantly associated with the OS (overall survival) of HCC. Moreover, multivariate analysis identified that the risk score served as an independent predictor for HCC patients (Fig 3F). These results indicated that necroptosis-related signatures could serve as independent prognostic factors in clinical prediction.

### Clinical utility of the necroptosis-related signature

**Fig 4:**
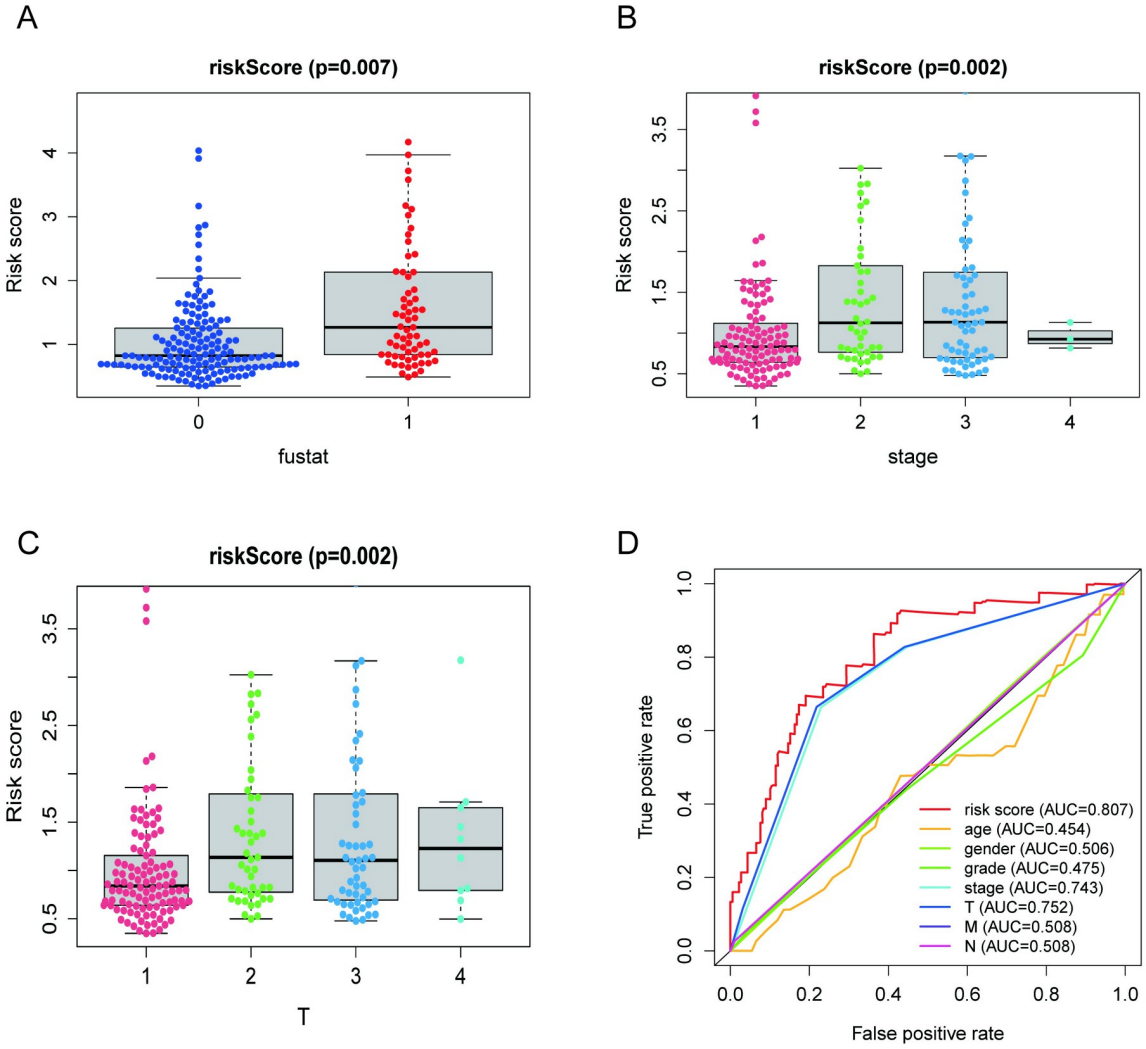
Clinical significance of the prognostic index. Risk score in (A) survival outcome, (B) tumor stage, (C) N stage.

We further analyzed the relationship between the NRG-based risk score and clinical characteristics (Fig 4A–4C). The results showed that the risk score was significantly associated with survival outcome (p = 0.007), advanced tumor stage (p = 0.002) and N stage (p = 0.002). We also analyzed the expression patterns of signature-related genes in clinical parameters (S1 Fig). In addition, ROC curves were generated to assess the predictive value of the 4 NRG risk signatures (Fig 4D) in the TCGA database. The area under the curve of the ROC curves was 0.807, which was significantly higher than for age (AUC = 0.454), sex (AUC = 0.506), grade (AUC = 0.475), stage (AUC = 0.743), T stage (AUC = 0.752), M stage (AUC = 0.508), and N stage (AUC = 0.508). Our research implies that the risk signature presented a better prediction value than clinical characteristics in HCC patients.

### Construction and validation of the nomogram

**Fig 5:**
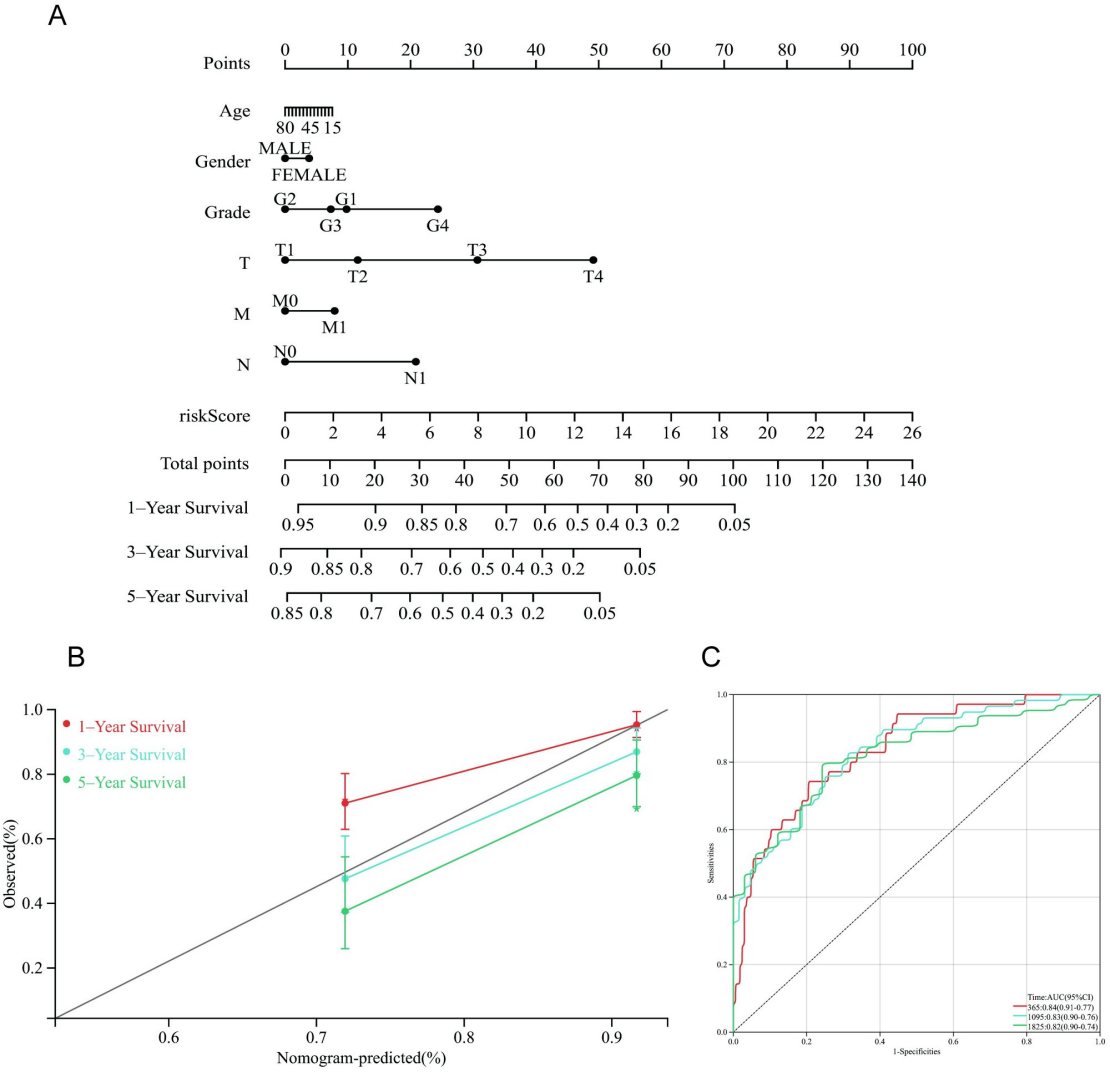
Drawing and validation of the nomogram. (A) 1-, 3-, and 5-year OS of HCC patients predicted by the nomogram. (B, C) The calibration plots and ROC curves for predicting 1-, 3-, and 5-year OS.

We established a nomogram to predict 1-, 3-, and 5-year OS in HCC patients based on the risk score and clinical parameters (age, sex, grade, stage, T stage, M stage, and N stage). The results are shown in Fig 5A. Meanwhile, the C-index, ROC curve, and calibration chart were used to evaluate the performance of the nomogram (Fig 5B and 5C). The C-index of the nomogram was 0.76, and the ROC curve showed that the AUCs for predicting 1-, 3-, and 5-year survival rates were 0.84, 0.83, and 0.82, respectively, which indicated better predictive value regarding survival. Moreover, the calibration chart indicated that the nomogram presented a high degree of consistency between prediction and actual observation.

### Validation of the necroptosis-related signature

**Fig 6:**
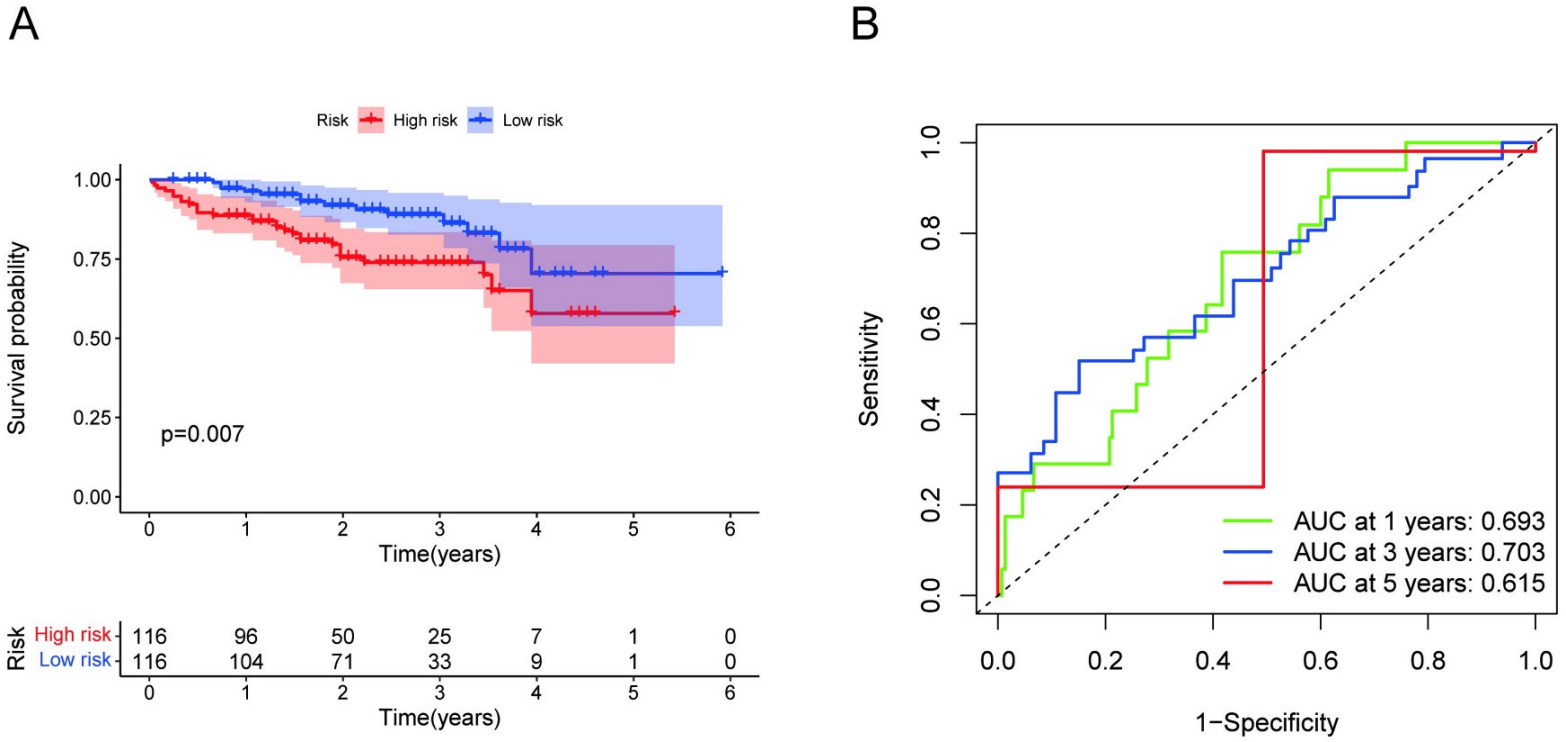
Validation based on the ICGC dataset. (A) Kaplan-Meier survival curves of patients in each group in the validation set. (B) ROC curves of 1-, 3-, and 5-year OS of patients in the validation set.

We calculated a risk score for each sample in another validation cohort based on the ICGC database using the same formula. According to the median risk score, the validation cohort was divided into high-risk and low-risk groups. As shown in Kaplan–Meier analysis, the OS rate of high-risk patients was significantly shorter than that of low-risk patients (Fig 6A). In addition, the AUCs of the ROC curves for the 1-, 3-, and 5-year survival rates were 0.693, 0.703 and 0.615, respectively (Fig 6B). As expected, our validation confirmed the remarkable value of the risk model constructed by the necroptosis-related signature in predicting the prognosis of HCC patients.

### Immunohistochemistry of the necroptosis-related signature

**Fig 7:**
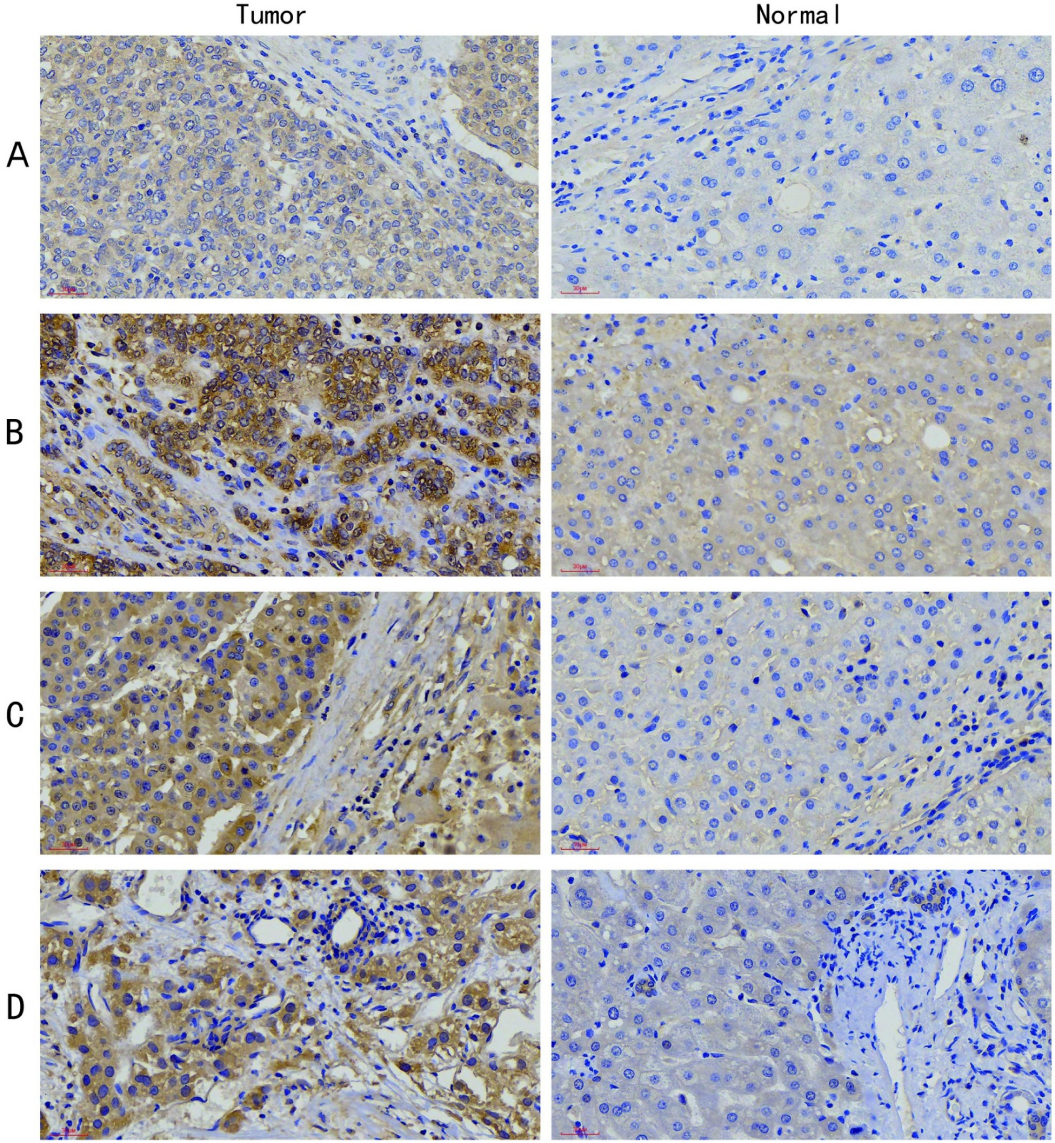
Representative images of immunostaining for HSP90AA1, PPIA, SQSTM1, and USP21 in liver tumor tissues and normal liver tissues. (A) Immunohistochemical analysis of HSP90AA1 expression, original magnification ×40. (B) Immunohistochemical analysis of PPIA expression, original magnification ×40. (C) Immunohistochemical analysis of SQSTM1 expression, original magnification ×40. (D) Immunohistochemical analysis of USP21 expression, original magnification ×40.

Immunostaining images of HSP90AA1, PPIA, SQSTM1, and USP21 in 20 tumor tissues and 10 liver specimens are displayed in Fig 7. The results revealed that the expression of HSP90AA1 (Fig 7A), PPIA (Fig 7B), SQSTM1 (Fig 7C), and USP21 (Fig 7D) was significantly increased in liver cancer tissues compared with normal liver tissues (p < 0.05, S2 Table), which was consistent with the results of the bioinformatics analysis.

### Prediction of response to immunotherapy and identification of potential chemotherapy drugs

**Fig 8:**
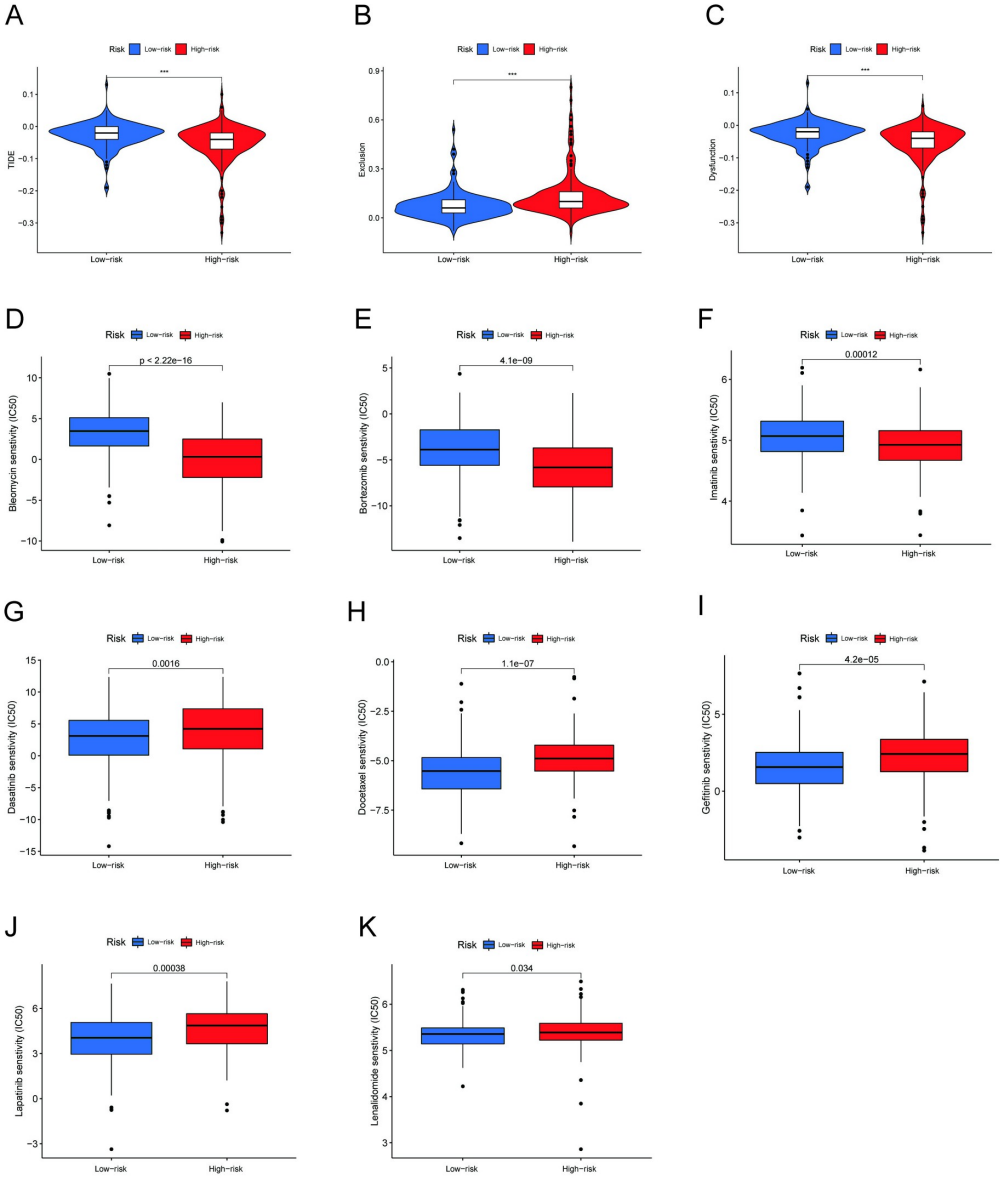
TIDE analysis and chemotherapy drug sensitivity in subgroups. (A-C) TIDE, T-cell exclusion and T-cell dysfunction score in different subgroups. (D-K) IC50 of common chemotherapy drugs.

The TIDE algorithm was used to evaluate the prospective effectiveness of immunotherapy in subgroups. According to our findings, the high-risk group had a lower TIDE score than the low-risk group, indicating that high-risk patients will respond to immune therapy better (Fig 8A). Additionally, we discovered that the high-risk group had higher T-cell exclusion scores and lower T-cell dysfunction scores (Fig 8B and 8C). Additionally, we examined treatment effect of chemotherapy in hepatocellular carcinoma based on the risk score (Fig 8D–8K). Bleomycin, bortezomib, and imatinib had relatively low IC50 values in the high-risk group, but dasatinib, docetaxel, gefitinib, lapatinib, and lenalidomide had higher IC50 values; attention to these results can be helpful in personalized treatment of high-risk group patients.

## Discussion

Hepatocellular carcinoma is a highly heterogeneous disease with a wide variation in survival times across patients with similar clinical features. However, clinical features alone could not provide accurate prediction of outcomes in patients with liver tumors. Recent studies indicated that novel prognostic models combined with gene signatures were used to optimize clinical decision-making [24, 25]. Necroptosis is a form of programmed cell death that has been reported to play a key role in tumor development, tumor necrosis, tumor metastasis, and tumoral immune response [26]. Dual effects of necroptosis on cancers have been reported. Key mediators of the necroptotic pathway can promote tumor progression, cancer cell invasion and metastasis in multiple cancers [16, 27]. However, Höckendorf, U. *et al*. reported that necroptosis could also protect against tumor development when apoptosis is impaired [28]. Although an increasing number of research articles have revealed the important role of necroptosis in tumor development and progression, a comprehensive analysis of NRGs has not been performed to investigate the clinical significance. In this study, we explored the relationship between necroptosis and hepatocellular carcinoma. Although similar studies are available, our study was validated with immunohistochemical experiments in 10 pairs of hepatocellular carcinoma and paraneoplastic samples [29].

First, we extracted the mRNA expression data of 159 NRGs in the TCGA-HCC cohort and then identified 36 differentially expressed NRGs based on the TCGA database. The GO analyses concluded that most of them were mainly involved in the regulation of I−kappaB kinase/NF−kappaB signaling and the regulation of the apoptotic signaling pathway. Park, S. *et al*. reported that receptor interacting protein 1 inhibits p53 induction through NF-kappaB activation and results in a worse prognosis in glioblastoma, which corroborated our functional enrichment analyses [30]. The KEGG analyses indicated that NRGs are mainly enriched in the shigellosis and NOD-like receptor signaling pathways in addition to necroptosis. Interestingly, the NOD-like receptor was found to be linked to neuronal necroptosis in cerebral ischemia-reperfusion injury [31]. Blériot, C. *et al*. also reported that Shigella can modulate epithelial cell necroptosis by activating a pathway involving Nod1 and RIPK2 [32].

To analyze HCC prognosis-related genes from the perspective of necroptosis, we identified 4 prognostic NRGs and confirmed the independent prognostic value of the necroptosis-related signature across univariate and multivariate Cox regression analyses. In previous studies, 4 necroptosis-related genes (HSP90AA1, PPIA, SQSTM1, and USP21) have been reported to be significantly associated with tumors. Xu, Q. *et al*. reported that HSP90AA1 promoted cell glycolysis and proliferation and inhibited apoptosis by regulating PKM2 abundance via Thr328 phosphorylation in hepatocellular carcinoma [33]. Cheng, S. *et al*. also reported that downregulation of PPIA promoted cell death and enhanced doxorubicin-induced apoptosis in hepatocellular carcinoma [34]. SQSTM1 was reported to inhibit hepatic stellate cell activity, fibrosis, and liver cancer by binding to the vitamin D receptor [35]. Our study showed that amplification is the most common genetic variant for USP21 in HCC. In addition, Hou, P. *et al*. identified frequent amplification of USP21 (22%) in human pancreatic ductal adenocarcinoma, and USP21 deubiquitinase promoted pancreatic cancer cell stemness via Wnt pathway activation [36]. Although there have been studies on hepatocellular carcinoma necrosis genes, validation by PCR alone is not sufficient [37]. In the present study, we observed high expression of HSP90AA1, PPIA, SQSTM1, and USP21 in HCC tissues by immunohistochemistry experiments, which indirectly validated the value of the necroptosis-related signature in HCC.

The Kaplan–Meier analysis indicated that the OS of the high-risk group was considerably shorter than that of the low-risk group. The risk score was also significantly associated with survival outcome, advanced tumor stage and N stage. Multi-index ROC curve analysis showed that the risk score (AUC = 0.807) presented better performance in prognostic prediction. We further developed a nomogram for the clinical prediction of 1-, 3-, and 5-year OS in HCC patients that combined risk scores and clinical parameters. Recently, it has been proposed that the use of Bayesian shrinkage models combined with multiple algorithms can effectively identify risk markers with good classification and prediction performance [38]. This algorithm was more suitable for high-dimensional variables than traditional algorithms, and the algorithm had computational efficiency. In the future, we will further classify the survival transformation and use Bayesian shrinkage models to improve the prediction performance of the whole model. The accuracy of the nomogram prediction was also verified by the C-index, ROC curve, and calibration. Recently, Mallick, H. *et al*. proposed performing differential expression analysis on transcriptome data using Tweedie models, an approach which is superior to the current common differential expression analysis methods in terms of statistical power and false discovery rate control [39]. Furthermore, Bayesian Shrinkage Priors in Zero-Inflated and Negative Binomial Regression models, which were derived from traditional models, had a feature of representing likelihood by a Polya-Gamma data augmentation; this offered the advantage of variable selection and lower mean square errors when compared to traditional regression models [40]. In future studies, we will further use this updated research method to perform the calculation of differential genes and variable selection and to fit high-dimensional data. We also conducted Kaplan–Meier analyses and ROC analyses to validate the value of the prognostic prediction signature based on an independent external cohort (ICGC-LIRI-JP), which demonstrated that the necroptosis-related signature could serve as an independent prognostic indicator.

The identification of patients who can benefit most from immunotherapy is vital and required since immunotherapy has been demonstrated to present significant potential in the treatment of HCC patients [41]. Therefore, investigating the molecular mechanisms of mRNA association with necroptosis that control immune evasion and immunosuppression may result in new immunotherapy strategies. TIDE is a cutting-edge computational technique that has gained recognition as a very accurate way to forecast how patients will respond clinically to immunotherapy [42]. Our data showed that low-risk patients had greater TIDE and T-cell dysfunction scores than high-risk patients, indicating that immune evasion and T-cell dysfunction may be associated with a poorer immunotherapy response. The high-risk patients also appeared to respond better to bleomycin, doxorubicin, gemcitabine, and lenalidomide. As a result, our work may offer a method for optimizing chemotherapy and immunotherapy regimens for individuals in the high-risk category.

However, there are several limitations in our study. First, retrospective data from public databases were used to establish and validate the prognostic model presented in this study. The clinical application should be verified by further prospective studies. Second, although we verified that four signature genes were highly expressed in liver cancer tissue samples by immunohistochemistry experiments, the exact molecular mechanisms need to be further explored.

## Conclusion

In conclusion, we established a prognostic risk model based on 4 necroptosis-associated genes (HSP90AA1, PPIA, SQSTM1, USP21) and validated it with an independent external cohort from the ICGC database and immunohistochemistry experiments. Our findings add new insight into clinical decision-making and provide new ideas for molecular targeted therapies.

## Supporting information

S1 Fig The univariate cox regression analysis. (TIF)

S1 Table List of necroptosis-related genes. (XLSX)

S2 Table Four NRGs expression. (DOC)

S1 File (RAR)

## Abbreviations

AUC: area under the curve
DEGs: differentially expressed genes
HCC: hepatocellular carcinoma
HSP90AA1: heat shock protein 90 alpha family class A member 1
ICIs: immune checkpoint inhibitors
NRG: necroptosis-related genes
PPIA: peptidylprolyl isomerase A
ROC: receiver operating characteristic
SQSTM1: sequestosome 1
TIDE: tumor immune dysfunction and exclusion
USP21: ubiquitin specific peptidase 21
