# Correction: Construction and validation of a prognostic signature based on necroptosis-related genes in hepatocellular carcinoma

**DOI:** 10.1371/journal.pone.0331773

**Published:** 2025-09-05

**Authors:** Yue-ling Peng, Ling-xiao Wang, Mu-ye Li, Li-ping Liu, Rong-shan Li

There is an error in affiliation 1 for authors Yue-ling Peng and Rong-shan Li. The correct affiliation 1 is: Department of Nephrology, Fifth Hospital of Shanxi Medical University (Shanxi Provincial People’s Hospital).
