# Supplementary figures and images for: Construction and validation of a prognostic signature based on necroptosis-related genes in hepatocellular carcinoma
Source: PLoS One. 2023 Feb 16;18(2):e0279744. doi: 10.1371/journal.pone.0279744 (PMC9934426; doi:10.1371/journal.pone.0279744)

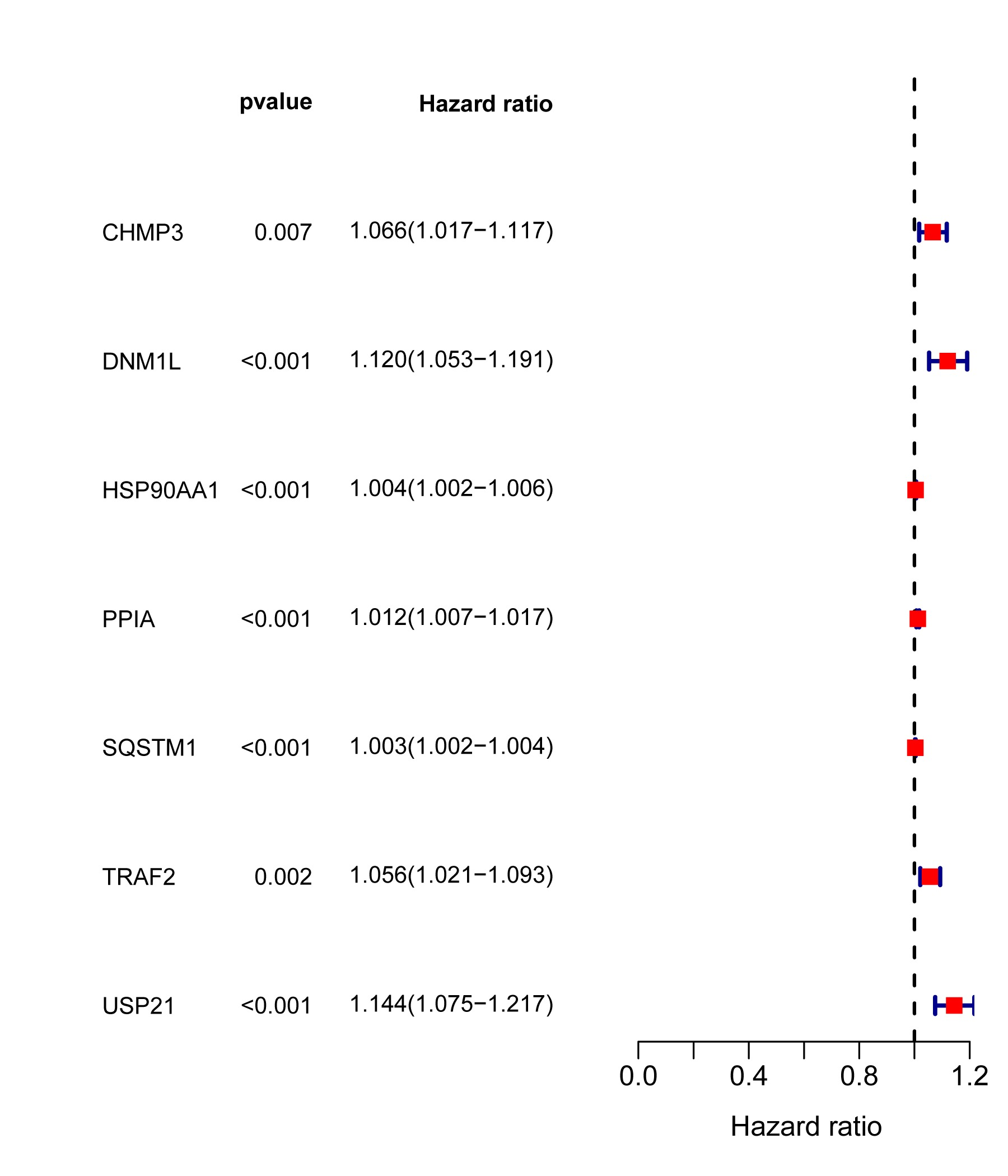

Supplement: S1 Fig — (TIF) [file pone.0279744.s001.tif]
