# Supplementary material for: Construction and validation of a prognostic signature based on necroptosis-related genes in hepatocellular carcinoma
Source: PLoS One. 2023 Feb 16;18(2):e0279744. doi: 10.1371/journal.pone.0279744 (PMC9934426; doi:10.1371/journal.pone.0279744)
Supplement: S2 Table — (DOC) [file pone.0279744.s003.doc]

Supplementary table 2: four NRGs expression in HCC tissues and normal liver tissues

HSP90AA1 expression in HCC tissues and normal liver tissues

|  | HSP90AA1 expression |  | P-value |
| --- | --- | --- | --- |
|  | positive | negative | P＜0.001 |
| HCC tissues | 17 | 3 |  |
| normal liver tissues | 0 | 10 |  |

PPIA expression in HCC and normal liver tissues

|  | PPIA expression |  | P-value |
| --- | --- | --- | --- |
|  | positive | negative | P＜0.001 |
| HCC tissues | 16 | 4 |  |
| normal liver tissues | 0 | 10 |  |

SQSTM1 expression in HCC and normal liver tissues

|  | SQSTM1 expression |  | P-value |
| --- | --- | --- | --- |
|  | positive | negative | P＜0.001 |
| HCC tissues | 17 | 3 |  |
| normal liver tissues | 0 | 10 |  |

USP21 expression in HCC and normal liver tissues

|  | USP21 expression |  | P-value |
| --- | --- | --- | --- |
|  | positive | negative | P＜0.001 |
| HCC tissues | 15 | 5 |  |
| normal liver tissues | 0 | 10 |  |
